# The impact of particulate matter 2.5 on the risk of preeclampsia: an updated systematic review and meta-analysis

**DOI:** 10.1007/s11356-020-10112-8

**Published:** 2020-08-01

**Authors:** Hongbiao Yu, Yangxue Yin, Jiashuo Zhang, Rong Zhou

**Affiliations:** Department of Obstetrics and Gynecology, Key Laboratory of Birth Defects and Related Diseases of Women and Children (Sichuan University) of Ministry of Education, West China Second University Hospital, Sichuan University, Chengdu, Sichuan People’s Republic of China

**Keywords:** Particular matter 2.5 (PM2.5), Preeclampsia, Meta-analysis, Pregnancy

## Abstract

There is increasing and inconsistent evidence of a linkage between maternal exposure to particulate matter 2.5 (PM2.5) and preeclampsia. Therefore, this study was conducted to investigate this relationship. Electronic databases including PubMed, Embase, Web of Science, and Cochrane Library were searched to identify articles published from inception to March 23, 2020, which showed a correlation between PM2.5 and preeclampsia. Finally, 9 of 523 initial studies were deemed eligible for inclusion. A random effect model was adopted to calculate the standardized odds ratio (OR) and 95% confidence interval (CI). Based on potential effect modification, subgroup analyses were further performed. Meta-analysis showed that maternal exposure to PM2.5 (per 10 μg/m^3^ increment) elevated the risk of preeclampsia (OR = 1.32, 95% CI 1.10 to 1.58%). Compared with other pregnancy trimesters, the third trimester of pregnancy seems to be the period in which women are more susceptible to PM2.5. Significant effect modification of the correlation between PM2.5 exposure and preeclampsia according to multiple pregnancies, pregnancy stage, maternal-related disease history, and sample size was not observed. The results demonstrated that maternal exposure to PM2.5 may predispose pregnant women to develop preeclampsia, especially in the third trimester of pregnancy. Therefore, more efforts should be made to improve air quality to maintain the health of pregnant women.

**Electronic supplementary material:**

The online version of this article (10.1007/s11356-020-10112-8) contains supplementary material, which is available to authorized users.

## Introduction

Preeclampsia is the most common pregnancy-related complication with no effective cure, which presents as a syndrome of elevated maternal blood pressure and/or proteinuria in pregnant women after 20 weeks of gestation (Shah 2006); it affects 3–7% of pregnancies in the world. Moreover, it is one of the major causes of increased maternal and fetal morbidity and mortality (Lyall et al. 2013; Steegers et al. 2010). The pathogenesis of preeclampsia is not fully understood. Placental dysfunction has been proved to be closely related to the pathogenesis of preeclampsia (Pierik et al. 2019). Some studies have proved that endothelial dysfunction and vascular remodeling failure were important factors for placental dysfunction (Blum et al. 2003; Sánchez-Aranguren et al. 2014; Valencia-Ortega et al. 2019). According to the immune abnormality theory of preeclampsia, pregnancy is a process of maternal immune adaptation to foreign objects (Milasinović et al. 2002). Once the maternal body can no longer tolerate the invasion of the trophoblast, it will impact the perfusion of the trophoblast, inducing ischemia and hypoxia of the trophoblast and releasing pro-inflammatory factors into peripheral blood (Ma et al. 2019). These primary changes can lead to a series of local and systemic effects, such as the formation of reactive oxygen species and activation of the maternal inflammatory and immune system (de Oliveira et al. 2010). These effects result in an imbalance between angiogenic factors and antiangiogenic factors, such as vascular endothelial growth factor (VEGF), placental growth factor (PlGF), soluble endoglin (sENG), and soluble fms-like tyrosine kinase-1 (sFlt-1), with predominance of the latter, eventually leading to placental vascular remodeling disorders and maternal systemic vascular endothelial dysfunction (Ramos et al. 2017; van den Hooven et al. 2012). At present, the etiology of preeclampsia is considered multifactorial and interactional. Genetic factors, dietary factors, psychosomatic status, behavioral factors, and other potential factors, such as increased particular matter 2.5 (PM2.5), may account for this condition (Adam et al. 2013; Cnattingius et al. 2004; Haelterman et al. 2003; Sun et al. 2020; Wang et al. 2015).

In recent years, worldwide industrial development and its accompanying air pollution have become a severe public health issue, especially the increase in PM2.5 (Anderson et al. 2012; Kampa and Castanas 2008). In 2006, PM2.5 was recommended by World Health Organization Air Quality Guidelines as an indicator of air particulate pollution (Feng et al. 2016). Statistically, 87% of the world’s population currently lives in environments with PM2.5 concentrations higher than the air quality standard of World Health Organization (Lippmann 2014). PM2.5 refers to fine particulate matter suspended in the atmosphere with an aerodynamic diameter less than 2.5 μm, which comes from a variety of sources, including road dust, motor vehicles, agricultural combustion, wood burning, waste incineration, and dust storms (Hasheminassab et al. 2014). The main components of PM2.5 include carbon, aluminum, lead, sulfur, bacteria, and other substances (Ibrahimou et al. 2014). PM2.5 can easily enter the respiratory tract, pass through the respiratory barrier, and disperse into the blood circulation because of its small diameter (Billet et al. 2007). Moreover, the complex components of PM2.5 may trigger a series of biological reactions, including oxidative stress, genotoxic injury, and immune and inflammatory responses, which produce acute or chronic injury to multiple organs and systems (Feng et al. 2016; Kreyling et al. 2002), leading to chronic obstructive pulmonary disease, diabetes mellitus, and pregnancy-related diseases (He et al. 2017; Melody et al. 2020; Xing et al. 2016).

Some studies have reported that atmospheric PM2.5 can enter the blood circulation and reach the maternal placenta causing or aggravating oxidative stress and inflammation, resulting in placental dysfunction and even preeclampsia (Brunst et al. 2018; Dadvand et al. 2013; Li et al. 2019; Slama et al. 2008). There is increasing evidence that PM2.5 exposure during pregnancy is positively associated with the risk of preeclampsia (Assibey-Mensah et al. 2020; Dadvand et al. 2013, 2014; Lee et al. 2013; Mandakh et al. 2020; Rudra et al. 2011; Wu et al. 2009); some studies have even suggested that PM2.5 exposure during a particular gestational period can increase the risk of preeclampsia (Lee et al. 2013; Mandakh et al. 2020). A systematic review and meta-analysis published 6 years ago assessed the correlation between PM2.5 exposure during pregnancy and preeclampsia but was limited by the small number of studies included and the lack of subgroup analysis for effect modification of this association (Pedersen et al. 2014). As the issue has been more widely explored, studies have challenged the positive correlation between exposure to atmospheric PM2.5 and preeclampsia as either absent or negative (Choe et al. 2018; Savitz et al. 2015).

Therefore, this meta-analysis aimed to review the published cohort studies assessing the effect of gestational PM2.5 exposure on preeclampsia to better understand the relationship between exposures to PM2.5 and preeclampsia and to provide evidence for maternal health protection during pregnancy.

## Methods

### Search strategies

The search filters were set as peer-reviewed original articles published in English from inception to March 23, 2020, which mentioned the correlation between maternal PM2.5 exposure and preeclampsia. The databases (PubMed, Embase, Web of Science, and Cochrane Library) were systematically searched with the following search terms: (“PM2.5” or “air pollutants, particulate” or “particulate matter” or “particulate air pollutants” or “traffic pollution” or “air pollution” or “indoor pollution” or “outdoor pollution” or “criteria air pollutant” or “fine particulate matter”) and (“Pre-eclampsia” or “preeclampsia” or “gestational hypertension” or “eclampsia” or “pregnancy toxemia” or “edema proteinuria hypertension gestos” or “hypertension in pregnancy” or “HELLP” or “pregnancy hypertension”). Furthermore, the reference lists of the included studies were manually retrieved to avoid omission.

### Selection criteria

The selection criteria for original literature were as follows. (1) Full text of the literature was available. (2) Studies were designed as epidemiological investigations in human subjects, including prospective cohort studies, retrospective cohort studies, or case-control studies. Time series studies, case reports, case series, systematic reviews meta-analyses, conference reports, lectures, or animal studies were excluded. (3) Pregnant women with a birth certificate were taken as the research population. (4) PM2.5 was taken as the air pollutant exposure factor. The definition of PM2.5 was clearly stated, the exposure concentration and increment of PM2.5 were provided, and the detection method (such as central air pollution monitoring station or residential monitoring) and calculation strategy (such as dispersion model or land-use regression model) of PM2.5 were described. (5) Maternal exposure to PM2.5 was clearly stated. The whole gestation period or a specific trimester was regarded as the exposure period: exposure periods other than pregnancy period were excluded. (6) The investigation outcome was preeclampsia or the subsets of preeclampsia, such as mild or severe preeclampsia, early- or late-onset preeclampsia, and eclampsia. Preeclampsia and its subsets were defined according to specific criteria, such as the International Classification of Diseases or the American College of Obstetricians and Gynecologists criteria. (7) Studies provided the odds ratio (OR) and 95% confidence interval (CI) or provided related data that can be changed into OR and 95% CI to describe the correlation between PM2.5 and outcome variables. (8) If two or more studies used the same population, the study with the largest sample size or the latest or the longest study period was included. (9) Studies were considered and adjusted for potential confounders that may affect the association.

### Data extraction

Endnote was used to export the literatures searched based on established search terms, and duplicates were automatically or manually removed. In reference to the abovementioned selection criteria, the titles and abstracts of retrieved literature were initially reviewed to screen for eligibility. Then, the remaining studies were further assessed by reviewing the full text. The following information, including first author, published year, study region, study design, sample size, period, exposure assessment method, exposure stage, and covariate adjustment, were extracted and are summarized in Table 1. Supplementary materials and relevant clues were traced when the main manuscripts lacked necessary data. If necessary, the authors of the included original studies were contacted for additional details.

**Table 1:** Main details of original studies included in this meta-analysis

Author	Publication year	Region	Study design	Total/case (*n*)	Period	Exposure assessment method	Exposure stage	Adjustment variables	Quality score
Wu et al.	2009	California, USA	Cohort	81,186/2442	1997–2006	Dispersion model	Entire pregnancy period	Study region, maternal race, poverty, prenatal care insurance type, infant sex, maternal age, parity, delivery type and method, and health conditions	7
Rudra et al.	2011	Washington, USA	Cohort	3509/117	1996–2006	Dispersion model	Entire pregnancy period	Maternal age, race/ethnicity, prepregnant BMI, smoking history and nulliparity, pregnancy season and year	7
Dadvand et al.	2013	Barcelona, Spain	Cohort	8398/103	2000–2005	Spatiotemporal	First, second and third trimester	Neighborhood socioeconomic status, ethnicity, education level, marital status, age, smoking, alcohol consumption, BMI, pregestational/gestational diabetes, parity, multiple pregnancy, season, and year of conception	8
Lee et al.	2013	Pittsburgh, USA	Cohort	34,705/1141	1997–2002	Personal monitor	First trimester	Maternal age, race/ethnicity, parity, smoked, season of birth, and year of conception	7
Dadvand et al.	2014	Barcelona, Spain	Cohort	3182/47	2003–2005	Personal monitor	Entire pregnancy period	Socioeconomic status, maternal ethnicity, education level, marital status, age, smoking during pregnancy, alcohol consumption during pregnancy, booking BMI, diabetes, parity, multiple pregnancy, gestational age at delivery, and season of conception	8
Savitz et al.	2015	New York, USA	Cohort	348,585/11166	2008–2010	Spatiotemporal adjustment model	First and second trimesters	Maternal age, race/ethnicity, education, conception year, BMI, and Medicaid status, identifying women of low income	8
Choe et al.	2018	Rhode Island, USA	Cohort	61,640/2221	2002–2012	Spatiotemporal model	Entire pregnancy period	Maternal age, parity, race, education level, marital status, health insurance status, and tobacco use during pregnancy, year of last menstrual period, and conditional on town of residence	7
Mandakh et al.	2020	Scania, Sweden	Cohort	35,570/1034	2000–2009	Spatiotemporal model	Entire pregnancy period	Maternal age, body mass index, parity, smoking, diabetes mellitus, gestational diabetes, essential hypertension, gestational hypertension, maternal country of birth, education level, annual household income, fetal sex, and year and season of birth	8
Assibey-Mensah et al.	2020	New York, USA	Cohort	16,116/732	2008–2013	Land-use regression model	Entire pregnancy period	Maternal age, race/ethnicity, education, parity, multifetal gestation, year of conception, prepregnant diabetes, gestational diabetes, prepregnant body mass index, birth hospital, relative humidity, and temperature	8

*Abbreviations*: *NOS* Newcastle Ottawa Scale, *BMI* body mass index

### Quality assessment

Two investigators (Hongbiao Yu and Yangxue Yin) used the combined criteria performed by Fan et al. (2015) and Shah et al. (2013) to independently assess the quality of the included original literature (Fan et al. 2015; Shah et al. 2013). Disagreements in the assessment were resolved by a third investigator (Rong Zhou). Thus, the quality of each study included in the meta-analysis was determined by the following metrics: generalizability, description of PM2.5, preeclampsia cases, source of preeclampsia data, reporting bias, limitations (the limitations mentioned in the original literature), multiple lag, adjustment for season or year of conception, adjustment for maternal epidemiological data (age, race/ethnicity, BMI, smoking, alcohol consumption, and parity, all should be included), and adjustment for funding support. Each of the above items received a score of 1; otherwise, a score of 1 was lost. The literature quality scores are summarized in Table 1.

### Statistical analysis

Stata 13.0 (StataCorp LLC, College Station, TX, USA) was used for all data processing, and statistical significance was nominally defined as two-sided and *P* < 0.05. The adjusted or converted ORs and 95% CIs of each included study were taken as the overall effect indicator. The correlation effect values extracted from the included studies were standardized by using the following formula (Shah et al. 2013) and considered 10 μg/m^3^ as the increased unit for atmospheric PM2.5. This means that if the concentration of PM2.5 increases/decreases by 10 μg/m^3^, the degree of OR and the 95% CI will increase/decrease:

$$ {\mathrm{OR}}_{\left(\mathrm{standardized}\right)}={\mathrm{OR}}_{\left(\mathrm{original}\right).}^{\mathrm{Increment}(10)/\mathrm{Increment}\left(\mathrm{original}\right).} $$

A random effect model was used to calculate the combined OR and 95% CI. Heterogeneity among included studies was estimated by using the *Q* test (Cochran 1954; Higgins and Thompson 2002). A *P* < 0.05 and *I*^2^ ≥ 50% indicates the existence of heterogeneity; the greater the *I*^2^ is, the greater the heterogeneity. To explore the source of heterogeneity and test the robustness of the correlation, sensitivity analysis was performed. The effect of individual studies on the whole risk estimation was also tested by a one-by-one elimination method. Subgroup analyses were further performed according to sample size, pregnancy stage, and whether maternal-related disease history and multiple pregnancies were excluded in the studies. The *Z* test was used to analyze the effect modification by sample size and whether maternal-related disease history and multiple pregnancies were excluded in the studies on the overall effect (Altman and Bland 2003). Univariate meta-regression analysis was used to analyze the effect modification by pregnancy stage on the overall effect (Altman and Bland 2003; Borenstein et al. 2009; Higgins and Green 2011; Hole et al. 2015; Thompson and Higgins 2002). If the *P* value of the effect modifier was less than 0.05, the subgroup effect was considered to be significant.

Egger’s test and Begg’s funnel plot were adopted to examine publication bias (Sun et al. 2020). If publication bias existed, a trim-and-fill method was adopted to further adjust the publication bias and to recalculate the combined risk estimates (Duval and Tweedie 2000).

## Results

### Characteristics of the included studies

**Fig. 1:**
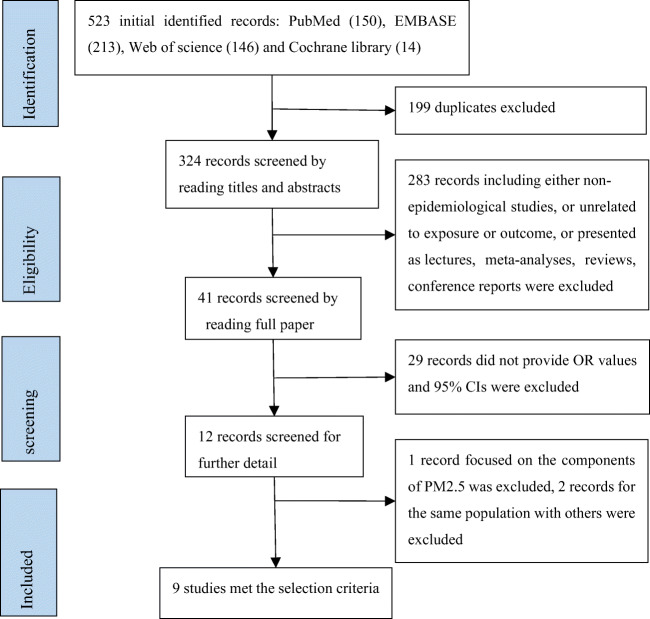
Flowchart of the selected literatures in this meta-analysis

The process of literature selection is summarized in Fig. 1. A total of 523 studies published in English from inception to March 23, 2020, were initially identified from the databases, including Cochrane Library (14), Embase (213), PubMed (150), and Web of Science (146). After eliminating duplicates, 324 studies remained. After reading the titles and abstracts of the studies, 283 studies, which were either nonepidemiological, unrelated to exposure or outcome, presented as meta-analyses, reviews, lectures, or conference reports, were excluded. By reading the full text of the remaining 41 studies, 29 studies without OR values and 95% CI, or other factors, were further excluded. Of the remaining 12 studies, 1 study was excluded because it did not describe the effect of PM2.5 on preeclampsia but rather the components of PM2.5, 1 study was excluded because it was a secondary analysis of a previous study, and 1 of 2 studies was excluded because it was conducted on the same population at different times. Finally, 9 studies ultimately met the selection criteria (Assibey-Mensah et al. 2020; Choe et al. 2018; Dadvand et al. 2013, 2014; Lee et al. 2013; Mandakh et al. 2020; Rudra et al. 2011; Savitz et al. 2015; Wu et al. 2009).

The main details of the final 9 studies are displayed in Table 1. All included studies were retrospective cohort studies. In terms of research region, 6 studies were conducted in the USA, 2 studies were conducted in Spain, and 1 study was conducted in Sweden. The studies were based on 3182 to 348,585 pregnant women, which occurred from 1996 to 2013 and comprised a total sample size of 592,891, among whom 19,003 had preeclampsia. The reported prevalence of preeclampsia ranged from 1.2% (Dadvand et al. 2013) to 4.5% (Assibey-Mensah et al. 2020). The definition of preeclampsia was not completely uniform across studies, i.e., some studies included eclampsia, some studies excluded superimposed preeclampsia, and some studies did not describe the definitions in detail (Table S1). Some studies excluded multiple pregnancies and maternal-related disease history (a history of hypertension, preeclampsia, or diabetes), while the others did not (Assibey-Mensah et al. 2020; Dadvand et al. 2013, 2014; Rudra et al. 2011). In addition to focusing on the correlation between PM2.5 exposure and the entire gestation period, some studies also divided the entire gestational period into three trimesters or nine gestational months to examine the association between PM2.5 exposure at different gestational stages and the risk of preeclampsia (Assibey-Mensah et al. 2020; Choe et al. 2018; Dadvand et al. 2013; Lee et al. 2013; Rudra et al. 2011; Savitz et al. 2015). Moreover, some studies further divided preeclampsia according to severity (Savitz et al. 2015), early-onset versus late-onset (Assibey-Mensah et al. 2020; Dadvand et al. 2013; Mandakh et al. 2020), or preeclampsia with versus without small-for-gestational age fetus (Mandakh et al. 2020).

Assibey-Mensah et al. (2020), Dadvand et al. (2013), Dadvand et al. (2014), Lee et al. (2013), Mandakh et al. (2020), Rudra et al. (2011), and Wu et al. (2009) observed that PM2.5 exposure was positively correlated with preeclampsia. Choe et al. (2018) and Savitz et al. (2015) indicated nonsignificant or infinitesimal association between PM2.5 and preeclampsia.

In addition, among the 9 studies, the PM2.5 exposure assessment methods were not entirely consistent (Table 1). The mean pregnancy PM2.5 exposure ranged from 10.1 (Rudra et al. 2011) to 16.5 μg/m^3^ (Dadvand et al. 2013).

### Correlation between PM2.5 exposure and preeclampsia

**Fig. 2:**
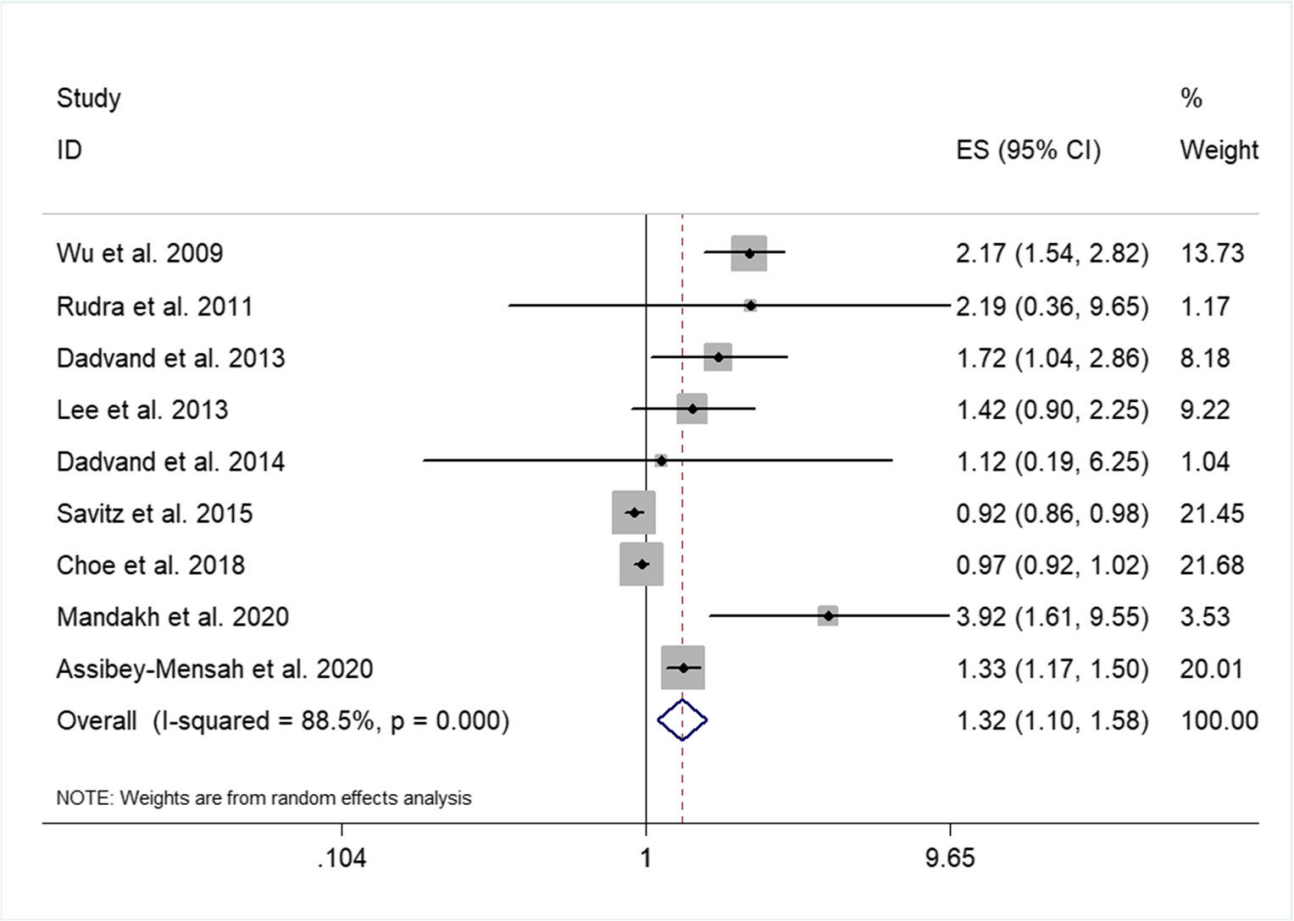
Forest plot of PM2.5 exposure and preeclampsia

To analyze the correlation between PM2.5 exposure and preeclampsia, the adjusted ORs and 95% CIs, which were extracted from the included studies, were incorporated into the algorithm. The calculated results were *I*^2^ = 88.5%, *P* < 0.001, indicating that heterogeneity exists (Fig. 2). The results of further analysis by random effect model showed that each 10 μg/m^3^ increase in PM2.5 concentration was significant in relation to preeclampsia, with an OR of 1.32 (95% CI 1.10 to 1.58%, *P* < 0.001), which showed a positive correlation between maternal exposure to PM2.5 and preeclampsia.

**Fig. 3:**
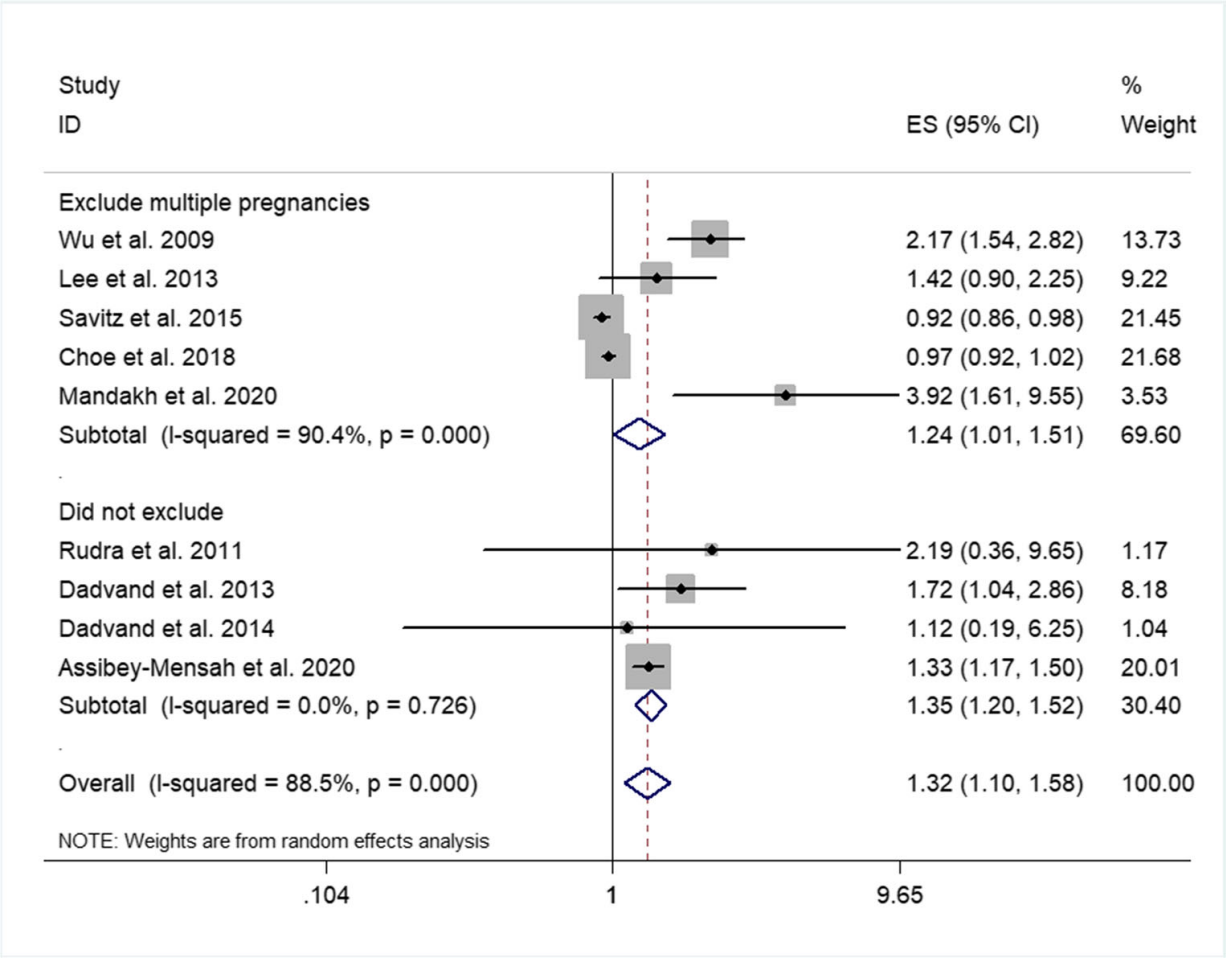
Forest plot of subgroup analysis on whether original studies excluded multiple pregnancies or not

**Fig. 4:**
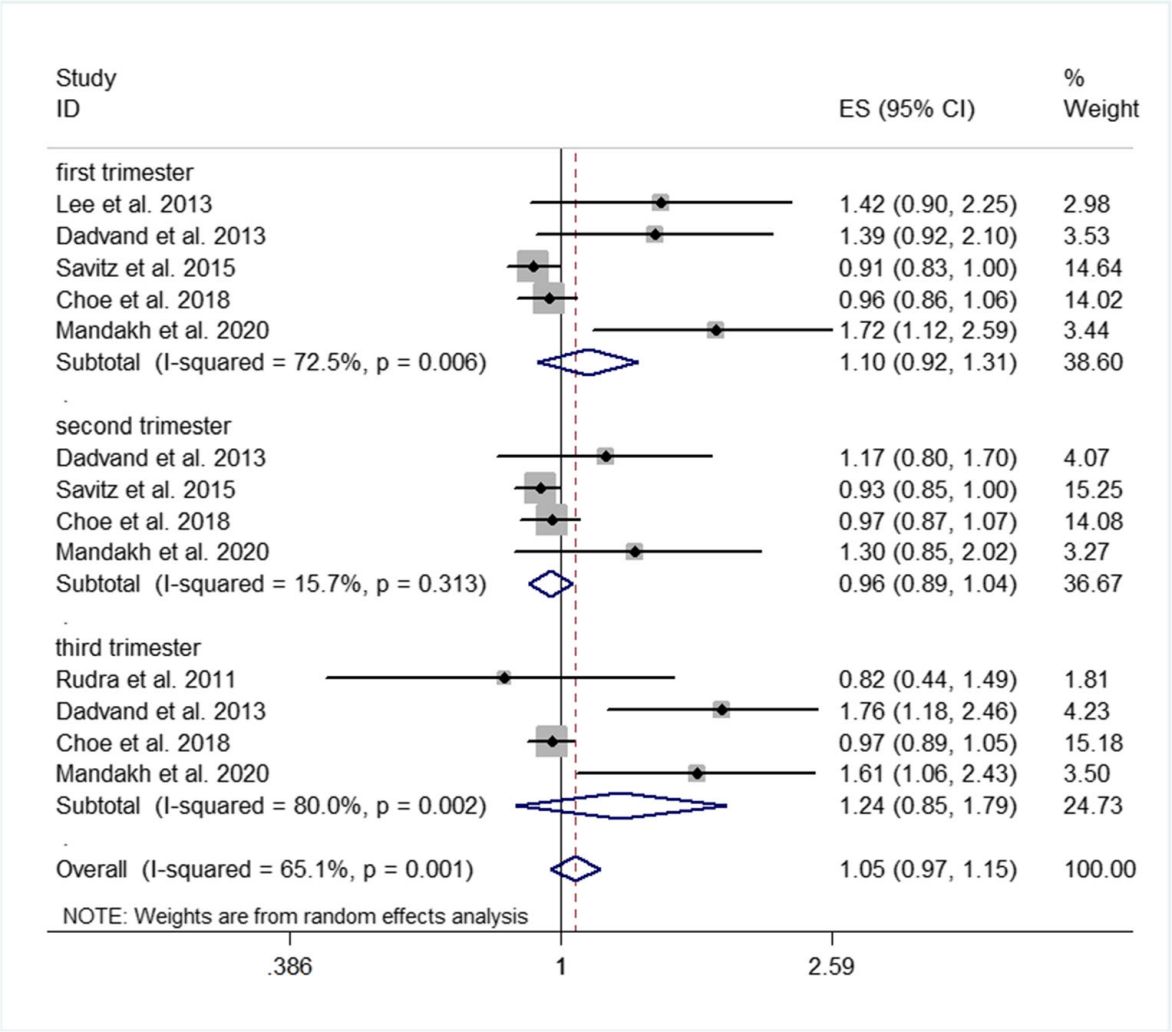
Forest plot of subgroup analysis on different gestational stages

Subgroup analyses were performed according to the potential effect modification by sample size, pregnancy stage, and whether maternal-related disease history and multiple pregnancies were excluded. The results showed that after excluding women with multiple pregnancies or maternal-related disease history, the correlation between PM2.5 exposure and the risk of preeclampsia was decreased than that before subgroup analysis but still positive (OR = 1.24, 95% CI 1.01 to 1.51%, *P* < 0.001) (Fig. 3; Supplemental Figs. S1). There was no significant difference between the subgroups excluding multiple pregnancies and those including multiple pregnancies or between the subgroups excluding maternal-related disease history and those including maternal-related disease history (*P* = 0.48 for both) (Table S2). After excluding the studies with sample size < 10,000, the results showed that the correlation between exposure to atmospheric PM2.5 and preeclampsia decreased than that before subgroup analysis but was still positive (OR = 1.28, 95% CI 1.05 to 1.54%, *P* < 0.001) (Fig. 4; Supplemental Fig. S2). There was no significant difference in the effect value between the subgroup with sample size < 10,000 and the subgroup with sample size >10,000 (*P* = 0.27) (Table S2). In addition, pregnant women were divided into three subgroups according to pregnancy stage. The results revealed that PM2.5 exposure in both the first and third trimesters of pregnancy was positively associated with preeclampsia (OR = 1.10, 95% CI 0.92 to 1.31%; OR = 1.24, 95% CI 0.85 to 1.79%; respectively), and it was more strongly correlated with the third trimester. Significant correlation between PM2.5 exposure in the second trimester of pregnancy and preeclampsia was not observed (OR = 0.96, 95% CI 0.89 to 1.04%), although there were no significant differences among the different pregnancy stage subgroups (first vs. second trimester, *P* = 0.57; second vs. third trimester, *P* = 0.42; first vs. third trimester, *P* = 0.76) (Table S2).

### Sensitivity analysis and publication bias

Sensitivity analysis of the included studies was carried out by using the one-by-one elimination method. The results showed that the combined values of OR (95% CI) and heterogeneity *P* were generally robust after sequentially excluding each study (Supplemental Figs. S3; Table S3).

Begg’s funnel plot had lack of notable symmetry, and Egger’s test result was significant (*P* < 0.05), suggesting publication bias (Supplemental Figs. S4, S5). A trim-and-fill method was used to adjust publication bias and to recalculate the combined risk estimates. The results showed that PM2.5 exposure was positively correlated with preeclampsia, which was significant (OR = 1.09, 95% CI 0.91 to 1.30%), yet heterogeneity still existed (*I*^2^ = 96.38, *P* < 0.01) (Supplemental Fig. S6).

## Discussion

This updated systematic review and meta-analysis summarized 9 cohort studies on the correlation between PM2.5 exposure during pregnancy and preeclampsia with a total of 592,891 observation subjects, among whom 19,003 had preeclampsia. The results showed that maternal exposure to elevated atmospheric PM2.5 concentration (per 10 μg/m^3^ increment) increased the risk of preeclampsia (OR = 1.32, 95% CI 1.10 to 1.58%, *P* < 0.001) (Wang et al. 2018). This meta-analysis also indicated that maternal exposure to PM2.5 (per 10 μg/m^3^ increment) in the third trimester of pregnancy was more likely to lead to the development of preeclampsia than exposure to PM2.5 (per 10 μg/m^3^ increment) in the other trimesters of pregnancy. Furthermore, this meta-analysis found no significant effect modification of maternal exposure to PM2.5 (per 10 μg/m^3^ increment) on preeclampsia by sample size (< 10,000), pregnancy stage, maternal-related disease history, and multiple pregnancies.

Preeclampsia is a serious pregnancy-related complication characterized by elevated maternal blood pressure and/or signs of damage to other organ systems after 20 weeks of pregnancy (Di Mascio et al. 2020). At present, the exact etiology of preeclampsia is not clear and is considered to be multifactorial and interactional (Pennington et al. 2012). Increasing attention to environmental pollution and mounting evidence shows that there is a significant correlation between air pollution and the risk of preeclampsia (Dadvand et al. 2013; Melody et al. 2020; Wu et al. 2009). PM2.5 in the air, in particular, has been confirmed to be positively correlated with the risk of preeclampsia according to increasing evidence from basic research, animal experiments, and epidemiological investigations (de Melo et al. 2015; Familari et al. 2019; Ibrahimou et al. 2014; Nääv et al. 2020). To date, the exact pathologic mechanism of PM2.5 and preeclampsia has been elusive. PM2.5 can easily enter the respiratory tract and disperse into blood circulation because of its small size (Billet et al. 2007). After entering the circulation, PM2.5 reaches the placenta and accumulates in the trophoblast cells. By causing damage to trophoblast mitochondria (Familari et al. 2019; Nääv et al. 2020), trophoblast vascular remodeling ability is reduced, and inflammatory factors and other biological mediators are released, which manifest as significant placental inflammation and increased systemic inflammation, oxidative stress, and immune response, leading to multiple organ damage, including severe placental dysfunction (Feng et al. 2016; Nääv et al. 2020; Kreyling et al. 2002). The latter manifests as the disruption of trans-placental oxygenation, decreased blood flow, and imbalance between angiogenic placental growth factors and antiangiogenic proteins, such as VEGF, PlGF, sFlt-1, and sENG (van den Hooven et al. 2012), and then preeclampsia ultimately develops. PM2.5 can also damage systemic vascular endothelial function and cause atherosclerosis (Auchincloss et al. 2008; Bo et al. 2016; Brook et al. 2010; Rundell et al. 2007). There is evidence that accumulated PM2.5 in trophoblast cells can cause changes in hormone level regulation (progesterone, human chorionic gonadotropin), which may produce damage to normal pregnancy and induce or aggravate the process of preeclampsia (Nääv et al. 2020). Moreover, the interaction between PM2.5 and the sympathetic nervous system alters the balance of the autonomic system and may account for this change (Hu et al. 2014).

Previous studies have shown that maternal PM2.5 exposure increases the risk of preeclampsia (Assibey-Mensah et al. 2020; Dadvand et al. 2013, 2014; Lee et al. 2013; Mandakh et al. 2020; Rudra et al. 2011; Wu et al. 2009), but other studies have challenged this positive correlation (Choe et al. 2018; Savitz et al. 2015). Of the 9 selected studies designed to assess a possible relationship between PM2.5 exposure during pregnancy and the risk of developing preeclampsia, 7 studies revealed a positive association. A retrospective cohort study of 81,186 pregnant women in Southern California revealed that exposure to PM2.5 during entire pregnancy increased the risk of preeclampsia (OR = 1.42, 95% CI 1.26 to 1.59%) (Wu et al. 2009). Rudra et al. (2011) showed a positive correlation between exposure to PM2.5 during entire pregnancy and the risk of preeclampsia (OR = 1.41, 95% CI 0.63 to 3.18%), but their study consisted of only 3509 women in Western Washington (Rudra et al. 2011). Dadvand et al. (2013) investigated 8398 pregnancies in Barcelona and observed an increased risk of preeclampsia associated with PM2.5 exposure during each trimester of pregnancy, with ORs (95% CI) of 1.29 (95% CI 0.94 to 1.76%), 1.12 (95% CI 0.85 to 1.48%), and 1.51(95% CI 1.13 to 2.01%) in the first, second, and third trimesters, respectively (Dadvand et al. 2013). Another study revealed that the increased odds of preeclampsia were correlated with PM2.5 exposure in a cohort of 34,705 pregnancies in Pittsburgh, PA (OR = 1.15, 95% CI 0.96 to 1.39%), but only the first trimester was observed (Lee et al. 2013). Based on data from 3182 pregnant women in Barcelona, Dadvand et al. (2014) found an OR 1.03 (95% CI 0.64 to 1.64%) for the association between PM2.5 exposure during entire pregnancy and risk of preeclampsia (Dadvand et al. 2014). Mandakh et al. (2020) proved an increased risk of preeclampsia associated with PM2.5 exposure during entire pregnancy, with an OR (95% CI) of 1.98 (95% CI 1.27 to 3.09%), according to investigation of 35,570 pregnant women in Scania, Sweden (Mandakh et al. 2020). Assibey-Mensah et al. (2020) revealed that PM2.5 exposure during entire pregnancy was associated with increased odds of preeclampsia (OR = 1.11, 95% CI 1.06 to 1.15%), according to an investigation of 16,116 pregnant women in New York (Assibey-Mensah et al. 2020). However, among 348,585 pregnant women in New York, Savitz et al. (2015) did not find evidence of an association between PM2.5 exposure during the first trimester of pregnancy and any risk of mild or severe preeclampsia (OR = 0.88, 95% CI 0.78 to 1.0%; OR = 0.95, 95% CI 0.82 to 1.1%, respectively), nor did it find evidence of an association between PM2.5 exposure during the second trimester of pregnancy and any risk of mild or severe preeclampsia (OR = 0.91, 95% CI 0.80 to 1.0%; OR = 0.96, 95% CI 0.81 to 1.1%, respectively) (Savitz et al. 2015). Choe et al. (2018) investigated 6164 women in Rhode Island and observed no increased risk of preeclampsia associated with PM2.5 exposure during each trimester of pregnancy, with ORs (95% CI) of 0.96 (95% CI 0.86 to 1.06%), 0.97 (95% CI 0.87 to 1.07%), and 0.97 (95% CI 0.89 to 1.05%) in the first, second, and third trimesters, respectively (Choe et al. 2018).

It should be noted that this systematic review and meta-analysis is an update of a previous study (Pedersen et al. 2014). It has several strengths. First, the present study includes a broader database search, more search terms, and longer literature publication period so that the scope of the literature search is more comprehensive, which can effectively avoid evidence omission. Second, this study contains more original literature than the abovementioned previous study. The newly added original studies included in this work introduced more cohorts, longer investigation periods, broader investigation areas, increased size in terms of the total samples and preeclampsia, and more confounding factors to adjust for pregnant women. The findings of 9 original studies on the correlation between PM2.5 exposure and preeclampsia were inconsistent: 7 studies were positive, and 2 were inconclusive. As a result, by including more investigation cohorts, samples, and extensive investigation areas, the research results will be more powerful. Third, this updated study shows that maternal exposure to PM2.5 (per 10 μg/m^3^ increment) is positively correlated with the risk of preeclampsia (OR = 1.32, 95% CI 1.10 to 1.58%, *P* < 0.001), and the previous study also showed that maternal exposure to PM2.5 (per 5 μg/m^3^ increment) is positively correlated with the risk of preeclampsia (OR = 1.31, 95% CI 1.14 to 1.50%, *P* < 0.001). Although the ORs (95% CI) of the two studies were similar, the increments were different. Therefore, the correlation of this study is lower than that of the previous study, but it still suggests that there is a positive correlation between PM2.5 exposure during pregnancy and the risk of preeclampsia. In addition, this study conducted subgroup analyses according to the potential effect modification by sample size, pregnancy stage, and whether maternal-related disease history and multiple pregnancies were excluded, which puts forward new research directions.

There is evidence that multiple pregnancies and maternal-related disease history (such as hypertension, diabetes, and preeclampsia) are important factors that increase the risk of preeclampsia (Duckitt and Harrington 2005; Mustafa et al. 2012; Steegers et al. 2010). Subgroup analysis was conducted based on whether the original study included multiple pregnancies or maternal-related disease history to assess the effect modification of these factors on the overall effect. The results showed that there was no significant difference in the effect values between the subgroups, suggesting that multiple pregnancies or maternal-related disease history did not modify the effect of PM2.5 exposure on preeclampsia. A study with small sample size may lead to the result deviating from the actual situation (Drazen et al. 2016). Subgroup analysis was performed based on whether the sample size of the included original study was less than 10,000 to assess the effect modification on the overall effect. No significant difference in the effect values between the subgroups was observed, which indicated that the sample size of the original study could not modify the effect of PM2.5 exposure on preeclampsia. The initial consideration of multiple pregnancies, maternal-related disease history, and sample size of the original study may be potential effect modifiers of the association between PM2.5 exposure and preeclampsia, but no significant effect modification was observed in the subgroup analysis results. The possible reasons include the limited number of original studies, the existence of studies that contradict the overall positive correlation, and the inconsistency in study design, exposure assessment, and outcome definition across the original studies (Higgins and Green 2011; Oxman and Guyatt 1992). More large-scale multicenter studies may solve this problem. On the other hand, the results of subgroup analysis showed that the effect values of all subgroups were greater than 1, whether including multiple pregnancies, maternal-related disease history, or sample sizes less than 10,000, which increased the reliability of the results of the overall analysis. Furthermore, subgroup analysis was performed based on different trimester of pregnancy: the results showed that there was no significant subgroup effect, suggesting that pregnancy stage may not be an effect modifier of the association between PM2.5 exposure and preeclampsia. The unequal number of original studies among the subgroups and the inconsistencies in study design, exposure assessment, outcome definition, and conclusions across the original studies may make it difficult to accurately detect subgroup effects (Higgins and Green 2011; Oxman and Guyatt 1992; Richardsona et al. 2019; Thompson and Higgins 2002). The results showed that PM2.5 exposure in the third trimester of pregnancy led to a higher risk of developing preeclampsia than PM2.5 exposure in other trimesters. The possible reasons are as follows: (1) During the progression of pregnancy, the uterus increases to the maximum height in the third trimester of pregnancy, making the diaphragm lift up and reducing the chest volume (relatively), and the maternal body exhibits a compensatory increase in tidal volume and minute ventilation volume (Kolarzyk et al. 2005); thus, the PM2.5 inhaled into the lung increases. (2) The high concentration of progesterone in the third trimester of pregnancy causes the smooth muscle of tracheobronchial tree to relax and promotes the decrease in total airway resistance (Bhatia and Bhatia 2000; Hu et al. 2014), allowing PM2.5 to enter the lungs more easily. (3) In the third trimester of pregnancy, pulmonary blood volume increases significantly with the increase in systemic blood volume (Hytten 1985), and the increase of progesterone level caused bronchiectasis and pulmonary mucosal congestion (Hegewald and Crapo 2011; Lomauro and Aliverti 2015). These changes made it easier for the PM2.5 inhaled into the lungs to be exchanged with the blood and enter the circulation. There is no exact explanation for the positive correlation between PM2.5 exposure in the third trimester of pregnancy and higher risk of preeclampsia, which needs to be further explored in future research. In addition, it must be stated that subgroup analysis was adopted to explore potential effect modification that is acceptable that, which to some extent, strengthened the relationship between factors and outcomes, but this approach may not be powerful enough and introduces some analytic challenges (Wang et al. 2007).

In this meta-analysis, there was heterogeneity in both the holistic analysis and the one-by-one elimination analysis. Nevertheless, all the analysis results showed OR > 1, so the heterogeneity was considered not to reflect the difference in correlation direction but the difference in magnitude. The heterogeneity may arise from differences in the study design, geographic locations, exposure estimate, definition of outcomes, and other potential factors. First, all included studies were retrospective studies, and there may be large selectivity bias and recall bias. Second, the included studies came from different countries and regions, and these different geographical locations may cause differences in temperature and humidity, economic status, and health management strategies, which may bring heterogeneity. Third, the measurement instruments and methods of PM2.5 in each study were not completely consistent. Some studies have used traffic-generated pollution data, air monitoring data, and particulate matter sample data, which have certain limitations. Due to the small space coverage, these data may only represent local pollutant data or reflect the change of pollutant level with time, so it is difficult to estimate the individual exposure, which will induce exposure bias. Though other studies have used computational models to assess air pollution, this approach still relies on environmental monitoring data and ignores the effects of different temperatures, humidity, and seasons on particle dispersion, which also increase the bias of PM2.5 exposure assessment. Most of the included studies were primarily focused on the effects of a variety of particulate matter (PM10, CO, NOx, and black carbon) in the atmosphere on preeclampsia. It is unclear whether other types of particulate matter affect the relationship between PM2.5 and preeclampsia. A study has shown that atmospheric PM2.5 is correlated with black carbon and delta-C (Assibey-Mensah et al. 2020). In addition, almost all of the studies did not analyze the components of PM2.5, such as aluminum, carbon, and barium, which ultimately affects pregnant women, while the components of PM2.5 may not be completely consistent across regions, which may also impact the results. The differences in land use, geographical location, traffic density, and composition in the included studies may also influence changes in air pollution exposure and may lead to different exposure biases. Furthermore, for exposure objects, all PM2.5 levels in the included studies were directly or indirectly derived from atmospheric pollutant monitoring results around residential areas during pregnancy, but pregnant women may spend more time indoors (working or living) or even use protective devices such as masks, so the PM2.5 levels provided may not fully represent the actual PM2.5 exposure. Almost all studies estimated PM2.5 exposure based on the birth sites, but the condition that pregnant women migrated to the birth sites near the end of pregnancy could not be ruled out, which may also lead to misclassification of PM2.5 exposure during pregnancy. Taken together, it might be more meaningful to use a personal monitor to measure maternal PM2.5 exposure per unit of time during pregnancy. Fourth, the included studies did not use a completely identical definition of preeclampsia, although blood pressure for preeclampsia was defined as ≥ 140 and/or 90 mmHg in all studies. However, other outcome definitions of preeclampsia were inconsistent, such as whether eclampsia or superimposed preeclampsia was included and whether proteinuria was included. Different outcome definitions and associated outcomes in individual studies may contribute to overall heterogeneity. If future studies can unify the diagnostic criteria, the results may be more valuable. Fifth, all studies were inconsistent in adjustment factors, and some of them ignored important adjustment factors, such as the prepregnancy BMI, education level, socioeconomic status, prepregnancy disease history, and maternal nutritional status, which have been proven to be risk factors for preeclampsia (Steegers et al. 2010), and may be potential confounders that cause information bias. Some studies have included delivery hospitals as an adjustment factor, while others have not: the confounding in delivery hospitals has been proven to affect the association between air pollution and preeclampsia, which may lead to confounding bias (Savitz et al. 2019). Sixth, most studies, with some exceptions, included a sample size of more than 10,000. Population heterogeneity may also amplify the potential for confounding. When the sample size is small, the sampling error is large, which may cause the result to be inconsistent with the actual situation. In contrast, when the sample size is large, the sampling error is small, and the result is closer to the real situation. Another source of heterogeneity is the differences in the extent of controlling effect modification, such as multiple pregnancies, prepregnancy disease history, and prenatal care. Although the exact source of heterogeneity was not found, the sensitivity analysis and trim-and-fill method verified the stability of the results. Therefore, more studies need to minimize heterogeneity to obtain more accurate correlation results.

### Strengths and limitations

This meta-analysis provided more accurate and robust statistical findings than individual studies on the positive correlation between elevated atmospheric PM2.5 concentrations and preeclampsia. Moreover, further analysis found that PM2.5 exposure in the first and third trimesters of pregnancy increased the risk of preeclampsia and that exposure in the third trimester was more sensitive.

This study has some limitations. First, heterogeneity was found in both the holistic analysis and the one-by-one elimination analysis of this study. Although possible sources of heterogeneity have been analyzed, this issue needs to be clarified and further explored in future studies. Second, although the included studies were based on a great number of cases, there were still relatively few studies on PM2.5 and preeclampsia. The meta-analysis included several studies that investigated only the correlations between exposure to PM2.5 and the first or third trimester of pregnancy, which may have introduced bias compared with PM2.5 exposure during entire pregnancy stage. Third, there was publication bias in the overall analysis. Although it had been adjusted with the trim-and-fill method, it could only be used as a statistical supplement and could not fully represent the real effect value. Fourth, many included studies have incorporated gestational hypertension, gestational diabetes, and preeclampsia as outcomes, and the interaction of these combined outcomes could not be ruled out. Fifth, the language of the included studies was limited to English; therefore, some studies published in other languages may have been omitted, which affects the sample size and statistical power. Finally, most of the included studies were primarily focused on the effects of a variety of particulate matter (PM10, CO, NOx**,** and black carbon) in the atmosphere on preeclampsia. It is unclear whether other particulate matters (such as noise and temperature) will affect the correlation between PM2.5 and preeclampsia.

Given the above limitations, the results need to be treated cautiously, and more large-scale multicentered studies are still required for clarification. It is suggested that future studies should optimize the study design, increase the sample size, improve the measurement methods and instruments of PM2.5, and standardize the outcome evaluation criteria to further explain this correlation and provide additional protection for pregnant women.

## Conclusions

The results of this meta-analysis showed that maternal exposure to PM2.5 (per 10 μg/m^3^ increment) increases the risk of preeclampsia. The results also indicated that maternal exposure to PM2.5 (per 10 μg/m^3^ increment) in the third trimester of pregnancy was more likely to develop into preeclampsia than exposure to PM2.5 (per 10 μg/m^3^ increment) in the other trimesters of pregnancy. In addition, this meta-analysis found no significant effect modification of maternal exposure to PM2.5 (per 10 μg/m^3^ increment) on preeclampsia by sample size (< 10,000), pregnancy stage, maternal-related disease history, and multiple pregnancies. Further research is needed to better understand the association and mechanisms between the maternal exposure to PM2.5 and the risk of preeclampsia.

Taking preventive measures to reduce the harmful effects of PM2.5 is necessary. First, the public should pay more attention to ambient air quality, strengthen the monitoring of PM2.5, and establish strict air quality assessment standards. Second, more active measures should be taken to reduce sources of PM2.5, such as reducing the burning of fossil fuels, garbage, and crops, decreasing industrial emissions, promoting the use of clean and renewable energy, and increasing green areas to reduce land dust. Furthermore, and more importantly, protections for pregnant women should be strengthened, especially in the third trimester, by reducing exposure time, using air purifiers and activated carbon filters that can absorb PM2.5, wearing dustproof masks, and maintaining adequate vitamin intake to help combat damage caused by PM2.5.

## Electronic supplementary material

ESM 1 (PDF 228 kb)

ESM 2 (DOCX 18 kb)

ESM 3 (DOCX 18 kb)

ESM 4 (DOCX 17 kb)
