# Supplementary material for: The impact of particulate matter 2.5 on the risk of preeclampsia: an updated systematic review and meta-analysis
Source: Environ Sci Pollut Res Int. 2020 Aug 1;27(30):37527–39. doi: 10.1007/s11356-020-10112-8 (PMC7496023; doi:10.1007/s11356-020-10112-8)
Supplement: Supplementary file 1 — (PDF 228 kb) [file 11356_2020_10112_MOESM1_ESM.pdf]

Supporting Figures for:

The impact of particulate matter 2.5 on the risk of preeclampsia: an updated systematic review and meta-analysis

Hongbiao Yu, Yangxue Yin, Jiashuo Zhang, Rong Zhou*

Department of Obstetrics and Gynecology, West China Second University Hospital, Sichuan University, Key Laboratory of Birth Defects and Related Diseases of Women and Children (Sichuan University) of Ministry of Education, Chengdu, Sichuan, China.

*Correspondence. E-mail address: zhourong_hx@scu.edu.cn; Tel: +8618180609085

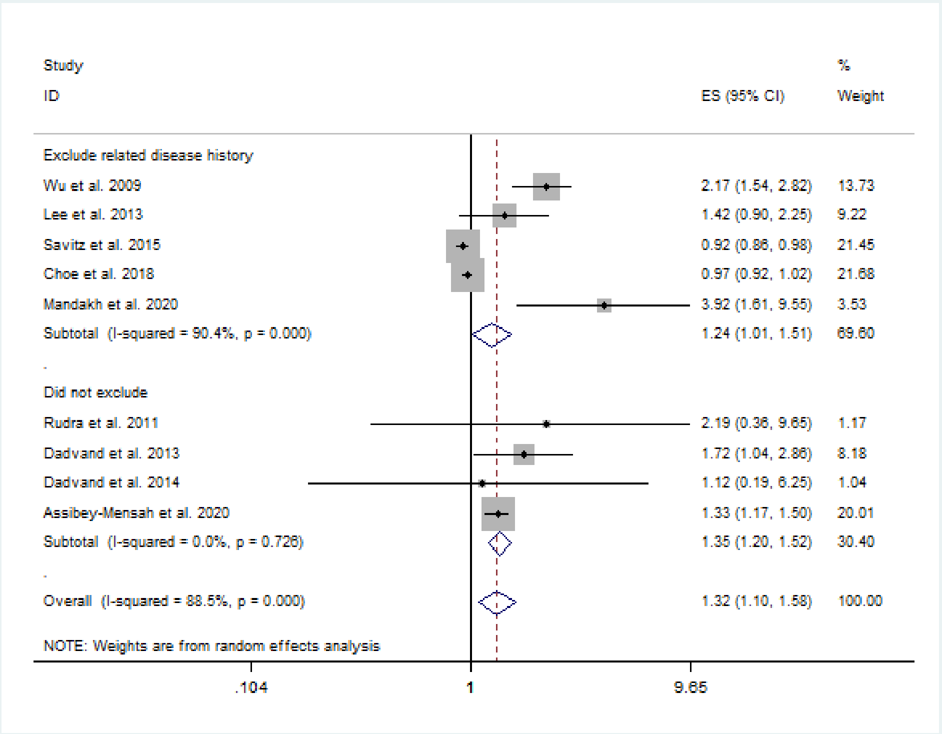

Fig. S1 Forest plot of subgroup analysis on whether original studies excluded maternal related disease history or not

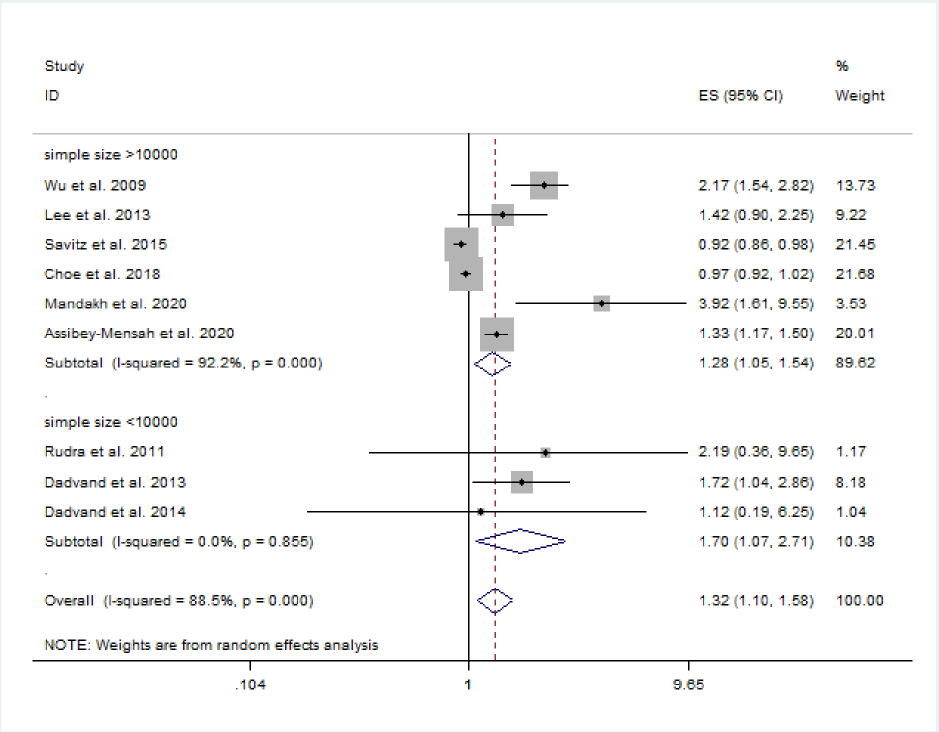

Fig. S2 Forest plot of subgroup analysis on whether the sample size of original studies >10,000 or not

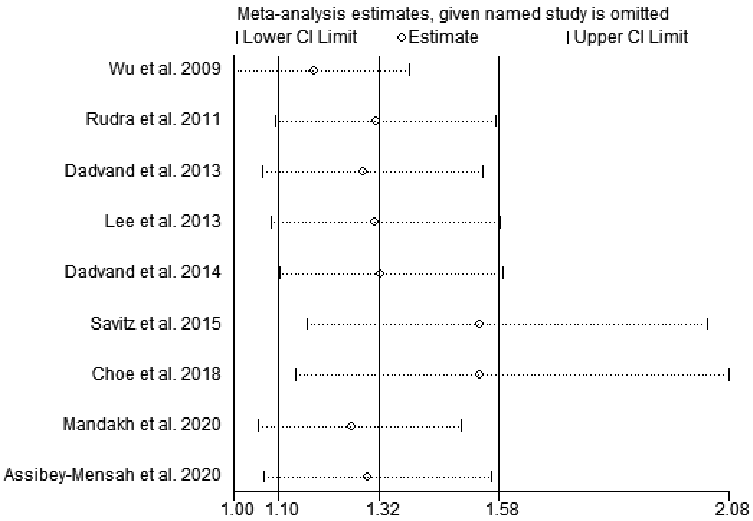

Fig. S3 Sensitivity analysis of exposure to $PM_{2.5}$ and preeclampsia

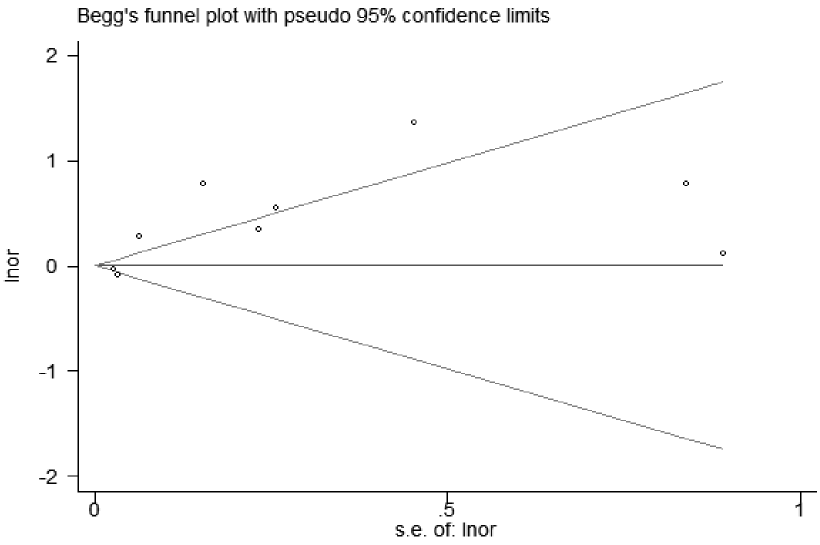

Fig. S4 Begg's funnel plot of exposure to PM2.5 and preeclampsia

```
Number of studies =  9                                  Root MSE      =   2.227
```

Std_Eff	Coef.	Std. Err.	t	P>|t|	[95% Conf. Interval]	
slope	-.0986608	.0575311	-1.71	0.130	-.2347002	.0373786
bias	2.631776	.9919651	2.65	0.033	.2861509	4.9774

```
Test of H0: no small-study effects          P = 0.033
```

Fig. S5 Egger's test of exposure to PM2.5 and preeclampsia

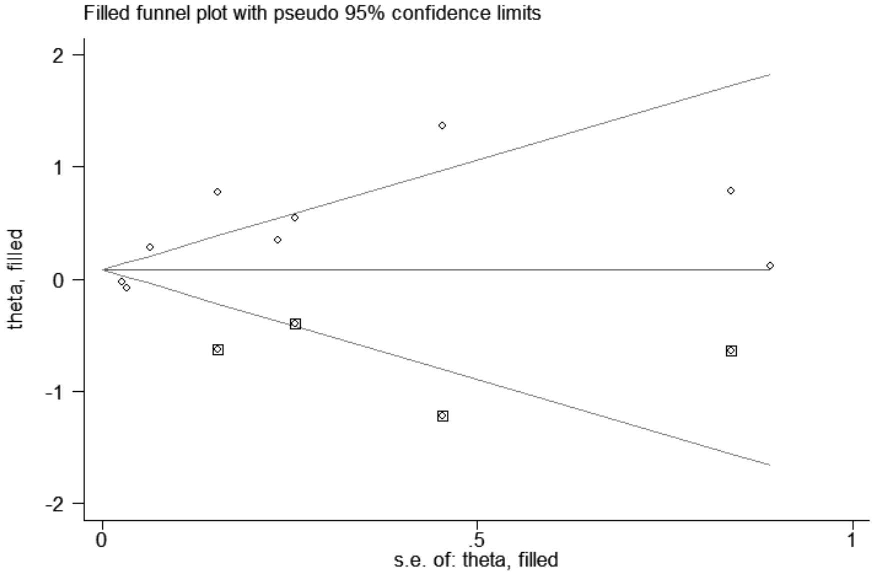

Fig. S6 Begg's funnel plot of exposure to PM2.5 and preeclampsia after performing trim and fill methods to adjust for publication bias
