# Supplementary material for: The impact of particulate matter 2.5 on the risk of preeclampsia: an updated systematic review and meta-analysis
Source: Environ Sci Pollut Res Int. 2020 Aug 1;27(30):37527–39. doi: 10.1007/s11356-020-10112-8 (PMC7496023; doi:10.1007/s11356-020-10112-8)
Supplement: Supplementary file 2 — (DOCX 18 kb) [file 11356_2020_10112_MOESM2_ESM.docx]

**Table S1 Preeclampsia definitions in the included studies**

| Author | Publication year | Preeclampsia definition |
| --- | --- | --- |
| Wu et al | 2009 | Preeclampsia defined as the occurrence of mild preeclampsia (blood pressure > 140/90 mmHg and proteinuria), severe preeclampsia (e.g., blood pressure > 160/110 mmHg and proteinuria with or without signs of end-organ involvement, including oliguria, liver function abnormalities, thrombocytopenia, headache), or hemolysis, elevated liver enzyme levels, and low platelet count (HELLP) syndrome at any time during pregnancy. Because hemolysis/HELLP is on the continuum of mild/severe preeclampsia and is relatively uncommon, we chose to combine this diagnosis with severe preeclampsia. |
| Rudra et al | 2011 | The criteria were sustained pregnancy-induced hypertension (≥ 140/90 mmHg) and proteinuria (urine protein concentrations of 30 mg/dL or 1+ on two or more urine dipsticks). |
| Dadvand et al | 2013 | Preeclampsia was defined according to the International Society for the Study of Hypertension in Pregnancy as resting blood pressure ≥ 140/90 mmHg on two occasions at least 4 hr apart and proteinuria ≥ 0.3 g/dL after the 20th week of gestation in previously normotensive women. Early-onset if diagnosed between weeks 20 and 34 of pregnancy, and as late-onset if diagnosed after week 34. |
| Lee et al | 2013 | Gestational hypertension was defined as systolic blood pressure (SBP) ≥ 140 mmHg or diastolic blood pressure (DBP)≥ 90 mmHg during the second half of pregnancy, whereas preeclampsia was defined as gestational hypertension accompanied by proteinuria after 20 weeks of gestation. |
| Dadvand et al | 2014 | Preeclampsia was defined according to the International Society for the Study of Hypertension in Pregnancy as resting blood pressure≥ 140/ 90 mm Hg on two occasions at least 4 h apart and the presence of proteinuria ≥0.3 g/dL after the 20th week of gestation in previously normotensive women. |
| Savitz et al | 2015 | Preeclampsia was defined by International Classification of Diseases, Ninth Revision, Clinical Modification (ICD-9-CM) discharge diagnoses: mild preeclampsia (ICD-9-CM codes: 642.40–642.44) and (4) severe preeclampsia/eclampsia (ICD-9-CM Codes:642.50–642.54 and 642.60–642.64). |
| Choe et al | 2018 | Preeclampsia was defined according to the presence of codes for either mild preeclampsia (642.40–642.44) or severe preeclampsia/eclampsia (642.50–642.64). |
| Mandakh et al | 2020 | Preeclampsia, excluding superimposed PE, is defined and diagnosed in accordance with the Swedish adaptation of the 10th version of the International Statistical Classification of Diseases and Related Health Problems (ICD-10) by the World Health Organization (WHO). |
| Assibey-Mensah et al | 2020 | Preeclampsia, defined in the electronic medical records using the International Classification of Diseases, Ninth Revision (ICD-9) discharge diagnoses of mild or unspecified preeclampsia (ICD-9 codes: 642.40-642.44), severe preeclampsia (ICD-9 codes: 642.50-642.54); or eclampsia (ICD-9 codes:642.60-642.64). |

**Supporting Information for:**

**The impact of particulate matter 2.5 on the risk of preeclampsia: an updated systematic review and meta-analysis**

Hongbiao Yu, Yangxue Yin, Jiashuo Zhang, Rong Zhou^*^

Department of Obstetrics and Gynecology, West China Second University Hospital, Sichuan University, Key Laboratory of Birth Defects and Related Diseases of Women and Children (Sichuan University) of Ministry of Education, Chengdu, Sichuan, China.

^∗^Correspondence. E-mail address: [zhourong_hx@scu.edu.cn](mailto:zhourong_hx@scu.edu.cn); Tel: +8618180609085
