# Supplementary material for: The impact of particulate matter 2.5 on the risk of preeclampsia: an updated systematic review and meta-analysis
Source: Environ Sci Pollut Res Int. 2020 Aug 1;27(30):37527–39. doi: 10.1007/s11356-020-10112-8 (PMC7496023; doi:10.1007/s11356-020-10112-8)
Supplement: Supplementary file 3 — (DOCX 18 kb) [file 11356_2020_10112_MOESM3_ESM.docx]

**Table S2 Subgroup analyses according to the potential effect modifications in the included articles**

| Subgroup | Included studies | OR (95% CI) | *P*- value for effect modification |
| --- | --- | --- | --- |
| Sample size |  |  |  |
| Sample size > 10,000 | (Wu et al. 2009; Lee et al. 2013; Savitz et al. 2015; Choe et al. 2018; Mandakh et al. 2020; Assibey-Mensah et al. 2020) | 1.28 (1.05-1.54) | 0.27 |
| Sample size < 10,000 | (Rudra et al. 2011; Dadvand et al. 2013; Dadvand et al. 2014) | 1.70 (1.07-2.71) |  |
| Maternal related disease history |  |  |  |
| Exclude maternal related disease history | (Wu et al. 2009; Lee et al. 2013; Savitz et al. 2015; Choe et al. 2018; Mandakh et al. 2020) | 1.24 (1.01-1.51) | 0.48 |
| Include maternal related disease history | (Rudra et al. 2011; Dadvand et al. 2013; Dadvand et al. 2014; Assibey-Mensah et al. 2020) | 1.35 (1.20-1.52) |  |
| Multiple pregnancies status |  |  |  |
| Exclude multiple pregnancies status | (Wu et al. 2009; Lee et al. 2013; Savitz et al. 2015; Choe et al. 2018; Mandakh et al. 2020) | 1.24 (1.01-1.51) | 0.48 |
| Include multiple pregnancies status | (Rudra et al. 2011; Dadvand et al. 2013; Dadvand et al. 2014; Assibey-Mensah et al. 2020) | 1.35 (1.20-1.52) |  |
| Pregnancy stages |  |  |  |
| First trimester | (Lee et al. 2013; Dadvand et al. 2013; Savitz et al. 2015; Choe et al. 2018; Mandakh et al. 2020) | 1.10 (0.92-1.31) | 0.57^a^ |
| Second trimester | (Dadvand et al. 2013; Savitz et al. 2015; Choe et al. 2018; Mandakh et al. 2020) | 0.96 (0.89-1.04) | 0.42^b^ |
| Third trimester | (Rudra et al. 2011; Dadvand et al. 2013; Choe et al. 2018; Mandakh et al. 2020) | 1.24 (0.85-1.79) | 0.76^c^ |

^a^ *P*-value for first trimester stratum compared with second trimester.

^b^ *P*-value for second trimester stratum compared with third trimester.

^c^ *P*-value for first trimester stratum compared with third trimester.

**Supporting Information for:**

**The impact of particulate matter 2.5 on the risk of preeclampsia: an updated systematic review and meta-analysis**

Hongbiao Yu, Yangxue Yin, Jiashuo Zhang, Rong Zhou^*^

Department of Obstetrics and Gynecology, West China Second University Hospital, Sichuan University, Key Laboratory of Birth Defects and Related Diseases of Women and Children (Sichuan University) of Ministry of Education, Chengdu, Sichuan, China.

^∗^Correspondence. E-mail address: [zhourong_hx@scu.edu.cn](mailto:zhourong_hx@scu.edu.cn); Tel: +8618180609085
