# Supplementary material for: The impact of particulate matter 2.5 on the risk of preeclampsia: an updated systematic review and meta-analysis
Source: Environ Sci Pollut Res Int. 2020 Aug 1;27(30):37527–39. doi: 10.1007/s11356-020-10112-8 (PMC7496023; doi:10.1007/s11356-020-10112-8)
Supplement: Supplementary file 4 — (DOCX 17 kb) [file 11356_2020_10112_MOESM4_ESM.docx]

**Table S3 Meta-analysis results after excluding studies one-by-one elimination**

| Studies excluded | Combined  OR (95%*CI*) | Heterogeneity | |
| --- | --- | --- | --- |
|  |  | *P*-value | *I^2^* |
| None | 1.32 (1.10–1.58) | < 0.001 | 88.5% |
| Wu et al. 2009 | 1.17 (1.00-1.38) | < 0.001 | 84.2% |
| Rudra et al. 2011 | 1.31 (1.09-1.57) | < 0.001 | 89.8% |
| Dadvand et al. 2013 | 1.28 (1.06-1.54) | < 0.001 | 89.3% |
| Lee et al. 2013 | 1.30 (1.08-1.58) | < 0.001 | 89.6% |
| Dadvand et al. 2014 | 1.32 (1.10-1.59) | < 0.001 | 89.9% |
| Savitz et al. 2015 | 1.53 (1.16-2.04) | < 0.001 | 88.3% |
| Choe et al. 2018 | 1.54 (1.13-2.08) | < 0.001 | 89.4% |
| Mandakh et al. 2020 | 1.25 (1.05-1.50) | < 0.001 | 88.4% |
| Assibey-Mensah et al. 2020 | 1.29 (1.06-1.56) | < 0.001 | 85.3% |

**Supporting Information for:**

**The impact of particulate matter 2.5 on the risk of preeclampsia: an updated systematic review and meta-analysis**

Hongbiao Yu, Yangxue Yin, Jiashuo Zhang, Rong Zhou^*^

Department of Obstetrics and Gynecology, West China Second University Hospital, Sichuan University, Key Laboratory of Birth Defects and Related Diseases of Women and Children (Sichuan University) of Ministry of Education, Chengdu, Sichuan, China.

^∗^Correspondence. E-mail address: [zhourong_hx@scu.edu.cn](mailto:zhourong_hx@scu.edu.cn); Tel: +8618180609085
